# TARGETING THE MTOR NETWORK TO MAXIMIZE HEALTHSPAN

**DOI:** 10.1093/geroni/igz038.1349

**Published:** 2019-11-08

**Authors:** Viviana I Perez

**Affiliations:** Linus Pauling Institute, Oregon State University, Corvallis, Oregon, United States

## Abstract

The mechanistic target of rapamycin (mTOR) is a key nutrient and growth factor-responsive pathway that has emerged as a central regulator of aging. Inhibition of mTOR complex I in particular has been found to increase lifespan and improve functional declines during aging in yeast, worms, flies, and mice. Recently clinical studies have provided the first evidence that similar effects may be achievable in pet dogs and in elderly people. This session will focus on new discoveries related to mTOR signaling and aging.

